# 

**Published:** 1857-08

**Authors:** 


					﻿No. III.]
AUGUST.
[Vol. XIII.
BUFFALO
MEDICAL JOURNAL
AND
lllontljh) lleuiciv
ar
MEDICAL AND SURGICAL SCIENCE.
SANFORD B. HUNT. NI. D.,
EDIT OK.
BUFFALO, N. Y.:
E. R. JEWETT & CO.’S STEAM PRESS.
Commercial Advertiser Buildings.
1857.
Price, $2.00 in advance, or $2.50 at the end of three month!
				

